# Mycotoxin Detection in Human Samples from Patients Exposed to Environmental Molds

**DOI:** 10.3390/ijms10041465

**Published:** 2009-04-01

**Authors:** Dennis G. Hooper, Vincent E. Bolton, Frederick T. Guilford, David C. Straus

**Affiliations:** 1 RealTime Laboratories, LLC, 13016 Bee Street #203, Dallas, TX 79234, USA; 2 5050 El Camino Real, #110, Los Altos, CA 94022, USA; 3 Department of Microbiology and Immunology, Texas Tech University Health Sciences Center, Lubbock, TX 79430, USA

**Keywords:** Aflatoxin, ochratoxin, trichothecene, human samples

## Abstract

The goal of this study was to determine if selected mycotoxins (trichothecenes, aflatoxins, and ochratoxins) could be extracted and identified in human tissue and body fluids from patients exposed to toxin producing molds in their environment. Human urine and methanol extracted tissues and sputum were examined. Trichothecenes were tested using competitive ELISA techniques. Aflatoxins B1, B2, G1, and G2, and ochratoxin A were tested by using immunoaffinity columns and fluorometry. Test sensitivity and specificity were determined. Levels of detection for the various mycotoxins varied from 0.2 ppb for trichothecenes, 1.0 ppb for aflatoxins, and 2.0 ppb for ochratoxins. Trichothecene levels varied in urine, sputum, and tissue biopsies (lung, liver, brain) from undetectable (<0.2 ppb) to levels up to 18 ppb. Aflatoxin levels from the same types of tissues varied from 1.0 to 5.0 ppb. Ochratoxins isolated in the same type of tissues varied from 2.0 ppb to > 10.0 ppb. Negative control patients had no detectable mycotoxins in their tissues or fluids. These data show that mycotoxins can be detected in body fluids and human tissue from patients exposed to mycotoxin producing molds in the environment, and demonstrate which human tissues or fluids are the most likely to yield positive results.

## 1. Introduction

Over the past 15–20 years, toxic mold exposure has become hazardous and frequent [1]. Scientific and medical literature has increased the awareness of mycotoxin producing fungi as possible pathogens in human disease [2,3]. After hurricane Katrina, the Center for Disease Control (CDC) issued new revised warnings as to the severe health effects of mycotoxin producing mold exposure [4]. Until now, there has been no readily available test to clinicians for helping establish the diagnosis of infections or illness due to toxin producing mold exposure. Currently, tests such as a sandwich enzyme linked immunosorbant assay (ELISA) test for antibodies to *Aspergillus* and polymerase chain reaction (PCR) for the detection of mold DNA in human specimens have been limited to clinical research investigating mold infections in immune-compromised patients (cancer, HIV, etc) [5,6]. There is a need to know not only if mycotoxin-producing molds are in the patient’s environment, but if these molds and their toxins are present in the patient, which could potentially be causing their medical problems.

Immunology tests such as competitive ELISA and immunoabsorbant (IA) columns utilize the concept of antigen-antibody reactions to capture and identify the antigen (a specific mycotoxin) by spectroscopy or fluorometry [7,8] and make it possible for the instant detection of mycotoxins from toxin producing or infectious molds.

Other work has demonstrated that mycotoxins can be detected in human sera [9–13] and in animal tissue [14]. Recently, work in China has been presented showing that plasma levels of aflatoxin B1 can be found in adolescents, which may impose substantial risk for hepatocarcinogenesis in adulthood [15]. Also, Tang [16] demonstrated that aflatoxin B1 can be determined in the serum. Ochratoxin A can also be measured in urine [17]. Additionally, trichothecenes can be found in urine [18]. However, our study is the first to examine large numbers of body fluids and tissue for the presence of mycotoxins in patients who have knowingly been exposed to environmental toxin producing molds. The findings presented in this study may also be helpful in identifying which tissues and/or fluids may be best for examination when trying to identify specific mycotoxins.

This study demonstrates that ELISA plates and IA columns can be adapted for human testing to evaluate the presence of mycotoxins. The mycotoxins studied were the aflatoxins, ochratoxins, and macrocyclic trichothecenes [19]. Validations were conducted in a CLIA (Clinical Laboratory Improvement Act of 1988) certified lab on patients with known exposure to mycotoxins or environmental molds and patients with no known exposure to the same. We demonstrate that competitive ELISA tests and IA columns can be used to evaluate tissues and fluids from patients who have been exposed to mycotoxin producing molds in the environment. The mycotoxin detection assays utilized specific mycotoxins to generate a qualitative determination of the presence or absence of fungal mycotoxins in clinical fluid and tissue.

## 2. Results and Discussion

In order to determine the limits of detection possible with our testing techniques a number of controls were developed. Validation results also tested accuracy, precision, level of detection (LOD), and interfering substances. Samples were also analyzed for matrix affect. Validations were conducted using CLSI (EP17 [20], EP5-A2 [21], and EP15-A2 [22]) specification. All criteria obtained were acceptable by CLIA standards. The “within-run” or “within-day” values all had standard deviations (SD) < 0.2. The “between-day” values all had reportable SDs of < 0.3. Interfering substances were not detected. Only level of detection (LOD) was determined in this study because this is a qualitative test only and actually obtained quantitative values are not reported. The importance of the quantitative amounts is yet to be ascertained and is not a part of these studies. The controls were samples of urine, nasal secretions, and tissue samples spiked with known amounts of the different mycotoxins being studied. Prior to spiking the samples, all samples were tested and demonstrated no detectable mycotoxins. As result of that testing and analysis, the lowest concentration of mycotoxin which could be detected was determined. Levels of detection of mycotoxins in human specimens reported in parts per billion (ppb) or ng/mL were determined to be > 1.0 for aflatoxins, >2.0 for ochratoxins, and > 0.2 for trichothecenes.

Specimens from patients with no known toxic mold exposures were tested to develop a set of reference data for a control group. The mycotoxin levels detected in the negative control group by specimen type is shown in Table 1. In urine samples, trichothecene levels were less that 0.2 ppb (mean equal to 0.08 ppb) and aflatoxin and ochratoxin levels were less than 1.0 and 2.0 ppb, respectively. In samples of nasal secretions, trichothecene levels were less than 0.2 ppb (mean equal to 0.12 ppb) while, the aflatoxin and ochratoxin levels were less than 1.0 and 2.0 ppb, respectively. In body tissues, trichothecene levels were less than 0.2 ppb (mean equal to 0.09 ppb) and again aflatoxin and ochratoxin levels were less than 1.0 and 2.0 ppb, respectively. All specimens were evaluated for the matrix effect (evaluating the components of a sample other than the analyte). Through the validation procedures, urine trichothecenes were diluted 1:5, while aflatoxins and ochratoxin A were diluted 1:7. Nasal secretions and tissue extracts showed no matrix affect.

Urinary data for all mycotoxins were calculated as nanograms per milliliter (parts per billion). Independent samples t-tests were performed on both the raw and log transformed datasets. The Mann Whitney U test, a non-parametric test, was also performed on both the raw and log transformed datasets. All data evaluated in all sets of mycotoxins showed a significant difference in levels by group (p<0.005).

The sensitivity and specificity of the various mycotoxin tests were determined, see Table 2. The sensitivity of our assay varied for both the type of mycotoxin and the sample being tested. For the detection of trichothecenes in urine, nasal secretions and body tissue, sensitivity varied from 44.4% to 94.5%. All samples were significantly different (p<0.005) from the control group. For aflatoxins in fluids and tissues, the sensitivity values varied from 17.4% to 70.6% (p<0.005) and for ochratoxin detection, the sensitivity values varied from 14.3% to 17.4% (p< 0.005). However, the specificity was 100% in all cases. The specimens were submitted by physicians in an attempt to rule mycotoxin exposure in or out. The symptoms acknowledged by physicians as being related to mycotoxin/mold exposure are considered by some to be vague. These include such symptoms as asthma, memory loss, fatigue, headache, muscle pains, or weakness. Many patients may carry a diagnosis of chronic fatigue syndrome, chronic asthma, muscular dystrophy, autism or dementia. Thus, a screening test may prove useful for patient care, especially if the test is negative. If a patient exhibits symptoms, the likelihood of having mycotoxin exposure increases (sensitivity) as can be seen in Table 2. Our data show that the likelihood of finding the toxins in human body tissues and/or fluids is greatest in urines for all toxins tested. The sensitivity for aflatoxins and ochratoxins is lower in all tissues. Our testing methods can detect those toxins (specificity) if they are present at the limit of detection.

Tables 3 and 4 demonstrate the aflatoxin and ochratoxin levels in patient tissue and body fluids. As can be seen from Table 3, the body tissue appears to be the best specimen to test when looking for aflatoxins. In body tissue, 35.7% of the samples were positive whereas nasal secretions and urine did not appear to be a good reservoir for aflatoxin detection. As can be seen in Table 4 the urine appears to be the best body fluid to test when looking for ochratoxin. In urine, 23.2% of the samples were positive, whereas nasal secretions and body tissues did not appear to be good reservoirs for ochratoxin detection.

Table 5 shows the trichothecene levels in specimens from patients exposed to mold and/or mycotoxins. As can be seen in this table that if trichothecenes are present in body fluids, urine appears to be the best body fluid to test. Trichothecenes were present in urines at levels between 0.2 and 1.0 ppb in sixty-two percent of the specimens. Although a small sampling is reported in this study, lung biopsies showed the presence of trichothecenes in significant numbers of specimens when compared to other tissue types. Other significant positive fluids were respiratory samples (e.g., sputa, BAL) and ear fluids. One spinal fluid was also positive for trichothecenes.

There have been no previous studies which have demonstrated large numbers of different tissues and/or body fluids to be contaminated with mycotoxins. This study shows that mycotoxins can be found in tissues and/or body fluids in humans after environmental exposure to toxin producing molds. The finding of mycotoxins in these fluids or tissues is consistent with clinical symptoms reported by these patients. Respirable trichothecene mycotoxins can be found in the air of *Stachybotrys chartarum* (SC) contaminated buildings [23,24,25]. The symptoms experienced by patients with the presence of mycotoxins include nausea, vomiting, ataxia, mental confusion, and variations in blood pressure. These symptoms are similar to those experienced by patients when a preparation of diacetoxyscirpenal (anguidine) [26] a simple trichothecene, was injected into humans. The symptoms experienced by patients in this study are also similar to the symptoms experienced by those individuals in SC infested buildings [27]. Brasel *et al.* [28] reported testing for airborne mycotoxins in non-agricultural settings by using competitive ELISA. It has been demonstrated that SC has been shown to grow in buildings where people are having health problems [28]. It has also been demonstrated that SC produces macrocyclic trichothecene mycotoxins in these buildings where these toxins are definitely inhaled by people [13]. These studies confirm that mycotoxins can be detected in human beings in concentrations sufficient to potentially cause the health problems observed in people in mold contaminated environments.

## 3. Experimental Section

### 3.1. Samples

Urine and nasal secretions were obtained from hospital patients or out-patients with a history of exposure to mycotoxins or fungi. Fixed autopsy and surgical biopsy specimens present in paraffin blocks from private autopsy specimens, pathology department surgical specimens, or coroner specimens were obtained from patients also with histories of exposure to mycotoxins or fungi. Negative control groups (Table 1) were the same type of specimens, but from patients with no known exposure to mycotoxins or molds. All specimens were placed into two groups. Group 1 was comprised of samples from individuals with no reported symptoms or known fungi or mycotoxin exposure. In this group, 55 urines and 27 upper respiratory (nasal washes and sputa) and 15 autopsy/surgical specimens were examined for the presence of mycotoxins (aflatoxins, ochratoxins, trichothecenes). Group 2 was comprised of samples from individuals with reported exposure to non-identified fungi or chemicals. Common symptoms of patients corresponding to group 2 samples included blurred vision, memory loss, fatigue, headache, nausea, loss of balance, cognitive deficits, rhinitis, sinusitis, rashes, and allergies. A detailed history and symptoms from many of the specimens were provided by many of the treating physicians (data not shown). Nasal secretions and washings were obtained by injection of 3–5 mL of sterile saline into each nostril of a patient. The patient was instructed to hold the saline in the nostrils for 30 seconds and then blow the saline into a sterile container held close to the nose. The specimen(s) were then collected and placed in containers.

### 3.2. Validation Samples

Validations were performed in a clinical laboratory for qualitative purposes only. Samples of extracted and filtered human heart tissue, liver tissue, urine, and nasal secretions (including sputum) were initially tested for the presence of mycotoxins (negative controls). All were negative for any type of mycotoxin tested. These negative control samples of mycotoxins were then spiked with different dilutions of aflatoxin (Sigma), ochratoxin (Sigma) roridin A (Sigma) and satratoxin H (Texas Tech University Health Sciences Center, Lubbock, TX). Samples were then evaluated for the amount of mycotoxin present. All samples were evaluated for the matrix effect by diluting the spiked samples in dilutions of 1:3, 1:4, 1:5, 1:6, and 1:10 amounts. Spiked amounts were evaluated as to the amount of known samples (mycotoxins) placed in each sample. Conclusions were then made as to which dilution for each mycotoxin should be used in the evaluation(s). These spiked amounts were used as calibrators to determine the levels of detection of each toxin. Each time a sample was evaluated, calibrators, negative, and positive, spiked tissues and fluids were also evaluated. Levels of detection were determined by guidelines established by the Clinical and Laboratory Standards Institute (NCCLS), document EP-17 A [26], which provide protocols for determining the lower limit of detection of clinical laboratory methods. The method in this paper is a qualitative validation, thus, actual values of specimens are not given. The EP-17 is intended for use by clinical laboratories for *in vitro* diagnostic tests. There is no claim for limit of quantification (LoQ) in this study. Validations were done as directed by the Clinical and Laboratory Standards Institute. Accuracy, precision, analytical sensitivity (detection limit) and analytical specificity (interfering substances) were the performance characteristics determined in this test. Accuracy was determined by running control samples and spiked samples of known amounts of mycotoxins 20 times within a designated run within a given day. Each material was also analyzed once per day for 20 days. Results reported were reported as standard deviations in terms defined as short term (within-run or within-day) experiment and long-term (between-day) experiment.

### 3.3. Preparation and evaluation of specimens for mycotoxin detection

Urine was received from a first-voided morning specimen and stored at 1–6 °C. In-house validations demonstrated that specimens could be held at 1–6 °C for seven days without affecting the results. Specimens were frozen at −20 °C for 24 months. A urine analysis was conducted using a dipstick to measure pH, specific gravity, glucose, nitrates, ketones, and blood. The urine was examined for sediment and was centrifuged at 2,500 g for 5 minutes (SeroFuge, Clay-Adams, Pesquady, NJ). if sediment was present. The pH of the urine was adjusted to 7.2 using a 1:5 dilution of urine-phosphate buffered saline (PBS) (pH 7.2) for the trichothecene testing. Nasal secretions and mucous samples as well as washes were observed for mucous presence. If mucous was present, a solution of MUCOSOL™ (Alpha Tec Systems, Inc. Vancouver, Washington) was prepared and added in equal amount of body fluid to Mucosol™ . After treatment, the sediment was re-suspended in PBS with a pH of 7.2. Tissue was received as either tissue fixed in a 10% formalin solution or in a paraffin-embedded tissue block. A minimum of 25–35 mg of formalin-fixed tissue was required for mycotoxin extraction. A maximum of 3 grams of formalin-fixed tissue was used if the tissue was in formalin less than three months. Tissue was placed into an autoclaved screw top tube containing silica bead beating glass. The specimens were lysed in a methanol/PBS solution optimized for the lysis of tissue and processed utilizing a bead-beater instrument (Biospec Products, Bartlesville, OK). The tubes were removed from the bead-beater and incubated to complete the lysis of the samples. After incubation, the samples were placed in 4.5 mL of PBS pH 7.2. Specimens were tested differently from this point to determine the presence of specific mycotoxins.

One mL of the prepared samples was applied to an IA column containing specific polyclonal antibodies directed against aflatoxins B1, B2, G1, and G2. The columns were washed twice with distilled water and then eluted with HPLC grade methanol (Sigma). One mL of a dilute bromine solution was added to the eluate, mixed, and read by fluorometry (Sigma, St. Louis, Missouri). Known standards of aflatoxin B1, B2, G1, and G2 were used (Trilogy Laboratories, Washington, MO) in 50 ppb, 25 ppb, and 2.5 ppb amounts.

One mL of the prepared samples mixed with an 80:20 mixture of methanol and water were applied to an IA column containing a specific monoclonal antibody to ochratoxin A. The columns were washed twice and eluted with a dilute solution of sodium hydroxide. The eluted samples were then read by fluorometry. Known standards of ochratoxin A were used (Trilogy Labs) at 50, 25, and 2.5 ppb.

Microtiter plates containing antibody to roridin A (a macrocyclic trichothecene) were used in this study. One hundred μL of calibrators with either satratoxin H or roridin A were added at concentrations from 0, 0.1, 1.0, and 10 ppb as standards for the curve determination. One hundred μL of urine, nasal secretions, or tissue extractions were added to wells. All samples and calibrators were done in duplicate. The specimens were then incubated for 15 minutes at 20–25 °C. Then 100 μL of enzyme horse radish peroxidase conjugated with a macrocyclic trichothecene was added and incubated for 15 minutes. Wells were washed to remove the unbound material with distilled water with Tween^®^ 20. One hundred μL of substrate was then added and incubated for 30 minute at 20–25 °C. On the addition of substrate, a color developed, the intensity of which is proportional to the amount of toxinenzyme bound to the well; i.e., the color intensity decreases with increasing concentrations of the toxin in the sample. Samples were read by spectroscopy at 450 nm. Results of the readings were correlated to a standard curve which gives a result in parts per billion (ppb). Standards of roridin A and/or satratoxin H were used (Trilogy Labs and RealTime Laboratories, LLC, Dallas,TX). The limit of detection was determined by spiking samples with known amounts of trichothecene (either satratoxin or roridin) and determining the lowest concentration that is detected.

## 4. Conclusions

Mycotoxins, specifically trichothecenes, aflatoxins, and ochratoxins, can be detected in human tissue and body fluids in patients who have been exposed to toxin producing molds in their environment. The toxins can be best determined in urine as a screening qualitative test which can assist the physician to determine what the best mode of therapy would be.

## Figures and Tables

**Table 1.** Mycotoxin values (in ng/mL (ppb)) by specimen in negative control group.

		Trichothecenes	Aflatoxins	Ochratoxins
Specimen type	n			
Urine	55	< 0.2 ppb Mean: 0.08 ppb	< 1.0 ppb	< 2.0 ppb

Nasal Secretions	27	< 0.2 ppb Mean: 0.12 ppb	< 1.0 ppb	< 2.0 ppb

Tissue	15	< 0.2 ppb Mean: 0.09 ppb	< 1.0 ppb	< 2.0 ppb

**Table 2.** Sensitivity and Specificity of Mycotoxin Tests.

		Sensitivity	Specificity
Trichothecenes			
	Urines	94.5 %	100 %
	Nasal Secretions	44.4%	100 %
	Tissue	58.8 %	100%

Aflatoxins			
	Urines	70.6%	100 %
	Nasal Secretions	17.4%	100 %
	Tissue	40.0 %	100 %

Ochratoxins			
	Urine	17.4%	100%
	Nasal Secretions	ND a	ND
	Tissue	14.3 %	100%

a ND = not detected

**Table 3.** Specimen types tested for aflatoxins from patients exposed to molds and/or mycotoxins.

Specimen Types	Negative Specimens	Positive specimens ≥ 1.0 ppb (ng/mL)	Number of specimens tested
Urine	120	58	178
Nasal secretions and sputa	41	6	47
Tissue block	18*	10**	28
Other Ear fluid (1) Spinal fluid (2) Bronchial alveolar lavage (BAL) (1) Vaginal fluid (1)	5	1 (ear fluid)	7
Total:	184	75	260

* Brain (3), liver (4), lung (3), muscle (2), bone marrow clot (1), bladder (1), skin (3), ovary (1).

** Liver (4), brain (3), lung (2), skin (1)

**Table 4.** Specimen types tested for ochratoxin A from patients exposed to molds and/or mycotoxins.

Specimen Types	Negative Specimens	Positive specimens ≥ 2.0 ppb (ng/mL)	Number of specimens tested
Urine	96	29	125
Nasal secretions and sputa	26	1 (sputum)	27
Tissue block	19*	1 (renal cell carcinoma)	20
Other Ear fluid (1) Spinal fluid (2) BAL (1)	4	1	5
Total	145	32	177

* Bone marrow clot (1), brain (2), liver (5), lung (5), skin (4), ovary (1), muscle (1).

**Table 5.** Specimens types tested for macrocyclic trichothecenes from patients exposed to molds and/or mycotoxins.

Specimen types	Negative Specimens	Positive specimens ≥ 2.0 ppb (ng/mL)	Number of specimens tested
Urine	223	437 #	660
Nasal secretions (includes sputa, nasal washes, BAL)	39	24	63
Tissue	18 *	14 **	32
Other	5^	9^^	14
Total:	285	475	769

# Sixty-two percent of all positives show 0.2 − 1.0 ppb (ng/mL) in urine.

* Bladder (1), ovary (1), brain (3), muscle (3), bone marrow clot (1), sinus biopsy (3), liver (3), and skin (3).

** Sinus (2), lung (7), brain (2), skin (1), liver (1), ovary (1).

^ Spinal fluid (2), feces (1), breast milk (1), vaginal secretions (1).

^^ Ear fluid (2), sputum (4), spinal fluid (1), BAL (1).
